# An Epidemic of Labial Chancres

**Published:** 1911-10

**Authors:** 


### An Epidemic of Labial Chancres

J. F. Schamberg, M.D., of Philadelphia, reports a series of nine cases, referred to a young man and transmitted by kissing games, at one or both of two parties. All but one of the cases occurred in girls and were apparently due to direct transmission. The other occurred in a man but must have been due to indirect transfer of the same virus, from the lips of one or more of the girls, not to contagion from a developed female case. One of the eight female cases was not labial but occurred on the cheek and was due not to kissing but, apparently to some other indirect transmission from two of the female patients who worked in the same office with her.

The two parties occurred, March 4 and 18, respectively and all of the cases developed during April, some early in the month.

The epidemic is not without its ludicrous side, especially as Philadelphia has the name of being a slow town but there is very little comedy and many, many years of tragedy not only for the nine victims and the remorseful originator of the epidemic, but for no one knows how many persons affected by an endless chain of infection, including the still-born, short lived and viable but more or less diseased offspring.

Is it not time that we realise that we have passed the period of healthy, intimate, neighborhood life when promiscuous kissing was an innocent diversion, or a mere friendly greeting, and when its physical dangers, less than at present in fact, were not appreciated? Society is a good deal more complex than it was a generation or two ago and, entirely aside from danger of infection with many diseases, the promiscuous exchange of carresses, however minor and innocent in themselves, is liable to lead to loss of mutual respect. It is a safe rule to start the anti-kissing rule in infancy, when strangers do not want to kiss the baby, and to keep it up.—Jour. A. M. A.

